# Aortocaval Fistula: A Rare Cause of Venous Hypertension and Acute Renal Failure

**DOI:** 10.1155/2012/487079

**Published:** 2012-12-30

**Authors:** Chandandeep Takkar, Lorraine Choi, Nasim Mastouri, Pradeep V. Kadambi

**Affiliations:** ^1^Division of Nephrology and Hypertension, Department of Internal Medicine at UTMB, University of Texas Medical Branch, 301 University Boulevard, Galveston, TX 77555, USA; ^2^Holzer Clinic, 100 Jackson Pike, Gallipolis, OH 45631, USA

## Abstract

Spontaneous rupture of abdominal aortic aneurysm into the inferior vena cava is rare and is associated with high mortality and morbidity. The clinical presentation can be variable and thus the diagnosis can be difficult. It can present with symptoms and signs of an abdominal emergency, venous hypertension, or systemic hypoperfusion. The traditional method of repair has been open surgery which is associated with high rate of complications. We report a case of aortocaval fistula (ACF) presenting with acute renal failure and heart failure, which was treated successfully with a novel, endovascular approach.

## 1. Case Presentation 

A 63-year-old Caucasian male with no known past medical history, presented with shortness of breath, productive cough, orthopnea, paroxysmal nocturnal dyspnea, and lower extremity edema of one month's duration. Patient also reported an associated weight gain of 7 pounds and occasional flank pain. Prior to admission, he had received treatment with azithromycin and levofloxacin for presumed community acquired pneumonia without improvement. Patient denied a history of smoking.

On admission, the vital signs were as follows: blood pressure 127/61, heart rate 101 beats/min, and regular, respiratory rate 18/min and oxygen saturation 98% on 2 liters/min of supplemental oxygen. Patient exhibited basilar pulmonary rales, a gallop rhythm, peripheral edema and elevated jugular venous pressure, suggestive of acute decompensated heart failure. Additionally, a loud abdominal bruit was heard and no abdominal masses were palpable. Pertinent laboratory data on admission included a creatinine of 1.6 mg/dL and hemoglobin of 11.6 g/dL. Urinalysis did not reveal hematuria or pyuria. 

**Figure 1:**
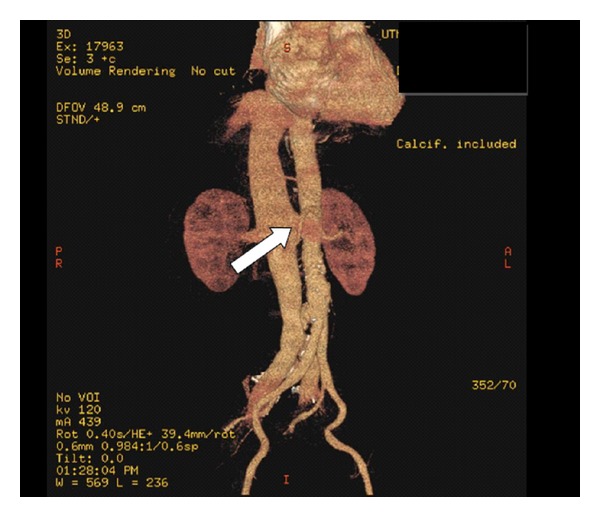
Arrow points to the fistulous connection between the aorta and the inferior vena cava.

He was started on medical management for acute decompensated heart failure. A transthoracic echocardiogram was performed which showed normal left ventricular systolic function, elevated right ventricular systolic pressure (RVSP > 60 mmHg) along with right ventricular hypertrophy. A diagnostic left and right heart catheterization was performed which showed 80% stenosis of the left anterior descending artery, needing angioplasty. In addition, there were findings of severe pulmonary hypertension, high cardiac output, and a stepup in oxygen saturation in the inferior vena cava (IVC) compared to that of superior vena cava (SVC). These findings are summarized in Tables 1 and 2. Subsequently, the patient developed hypotension necessitating transfer to intensive care unit for vasopressor support. He rapidly developed signs of visceral and peripheral hypoperfusion including acute hepatic failure, oliguric acute renal failure, and digital ischemia. Continuous renal replacement therapy (CRRT) was required for the management of complications of acute renal failure. Patients' blood cultures remained negative. The unclear etiology of cardiogenic shock, an abdominal bruit, and elevated IVC oxygen saturation prompted us to suspect an infradiaphragmatic fistulous connection between the arterial and venous system. A 3-dimensional CT angiogram was performed, which revealed an aneurysmal aorta (2.8 cm diameter) with diffuse atheromatous plaques and a 1.2 cm long, fistulous connection between distal aorta and IVC, above the level of the iliac bifurcation (Figure 1). A diagnostic aortogram was subsequently performed which revealed immediate filling of the contrast into the dilated IVC, confirming the diagnosis. The blood flow to both renal arteries was found to be normal during the procedure. The ACF was repaired by the placement of an excluder aortic endograft by a retrograde femoral approach.

**Figure 2:**
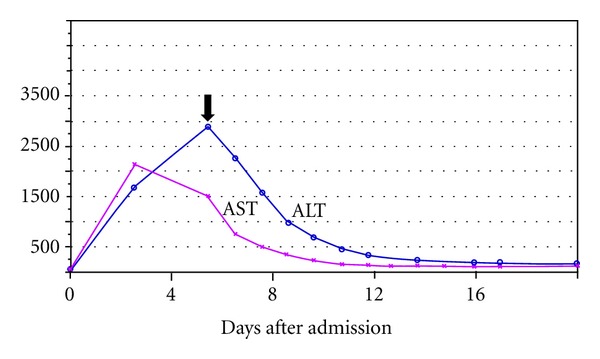
Trend of the liver enzymes (IU/L) after repair of the ACF (arrow).

Before the procedure, the venous pressure in SVC was 35 mmHg. Immediately following the closure of the fistula, there was a dramatic drop in SVC pressure to 14 mmHg. Subsequently, patient's hemodynamics improved and he was weaned off vasopressor support, liver enzymes declined with gradual return to normal values (Figure 2), and signs of digital ischemia improved. The patient however stayed dependent on intermittent, conventional hemodialysis due to nonrecovery of renal function. Patient was discharged to a long-term care facility. 

## 2. Discussion 

Spontaneous ACF is a rare complication of an abdominal aorta aneurysm. It was first described by Syme in 1831 [1] and the first successful repair was done by Cooley et al. in 1954 [2]. While majority of the cases of ACF are spontaneous, about 20% result from trauma or iatrogenic causes. ACF occurs in about 1% of patients with abdominal aortic aneurysms [3, 4] and the proposed mechanism involves severe periaortic inflammation leading to adherence of aneurysm to IVC. Death typically results without intervention in less than 2 months [5].

The clinical presentation of patients can be variable, either with signs of an abdominal catastrophe or those of cardiogenic shock or venous hypertension. Majority of patients reported to date are men in the sixth to seventh decade of life, with underlying risk factors for atherosclerotic disease such as smoking and hypertension [6]. The typical presentation, which may be present in less than 20% of patients, has been described as sudden onset of back pain, often with an abdominal bruit, a pulsatile abdominal mass, hematuria, bleeding per rectum, and systemic hypotension. In contrast, presenting symptoms may be subtle and rarely, it may be discovered in an asymptomatic patient undergoing diagnostic workup for an unrelated concern [7]. 

Presentation with signs and symptoms of high output cardiac failure as in our patient with or without signs of peripheral and visceral hypoperfusion has been reported as well. Acute renal insufficiency has been described in majority of patients presenting with this condition and is postulated to result from hypoperfusion resulting either from the shock state or severe venous hypertension [8]. The renal failure is potentially reversible, after closure of the shunt in most instances [9]. The intraprocedural hemodynamic measurements in our patient supported severe left to right shunting preoperatively with improvement of venous hypertension after closure of the shunt. We attributed the nonrecovery of our patient's renal function to multiple renal insults, including repeated contrast exposure, prolonged state of shock, and venous hypertension. 

Unusual presentations include a paradoxical pulmonary embolism from an aortic thrombus [10] and acute renal colic, with or without hematuria [11]. Also, increased myocardial oxygen demand following the spontaneous rupture of abdominal aortic aneurysm (AAA) into IVC may precipitate angina [12]. 

The diagnosis can be made preoperatively, in a stable patient, with ultrasonography with Doppler, CT angiography, or MRI angiography. In unstable patients the diagnosis might be made intraoperatively, or as in our patient, with a diagnostic/interventional aortography. 

Conventionally, treatment has been open surgical repair with its attendant complications, including up to 30% mortality rate [13]. In our patient, a novel, endovascular approach was utilized for the management. Advantages of endovascular approach include lesser intra-and perioperative complications such as blood loss and could therefore, be the preferred mode of treatment in patients without associated rupture of AAA and in those who are believed to be too unstable for an open surgery. The reported success rate of endovascular repair of abdominal arteriovenous fistulae is about 96% with no short-term mortality [14]. The most common complication related to the procedure is a type 2 endoleak, seen in about 22% patients [14]. 

Our patient differed from the reported cases previously in lacking the history of smoking, hypertension, diabetes, or other known risk factors for severe atherosclerotic disease. The size of AAA was also much smaller (2.8 cm compared with mean 11 cm in previously reported ACF). The management by endovascular rather than an open surgical approach is also a novel therapeutic option.

## Figures and Tables

**Table 1:** Hemodynamic measurements during diagnostic cardiac catheterization (pressures in mmHg and cardiac output in L/min). The numbers in brackets represent mean pressures.

	Pressures (mmHg)
Pulmonary artery	74/27 (47)
Pulmonary wedge	59/46 (32)
Right ventricle	82/1 (21)
Right atrium	41/45 (28)
Aorta	131/65 (93)
Cardiac output thermal	13.3 L/min
Cardiac output fick	16.6 L/min

**Table 2:** Oxygen saturation (%) measured during cardiac catheterization.

Pulmonary artery	89
Right ventricle	86
Right atrium	87
Superior Vena Cava	64
Inferior vena cava	93
Femoral artery	98
